# Blends of rABS and SEBS: Influence of In-Situ Compatibilization on the Mechanical Properties

**DOI:** 10.3390/ma12152352

**Published:** 2019-07-24

**Authors:** Zhiming Zhan, Hezhi He, Zhiwen Zhu, Bin Xue, Guozhen Wang, Ming Chen, Chengtian Xiong

**Affiliations:** 1 National Engineering Research Center of Novel Equipment for Polymer Processing, Guangdong Provincial Key Laboratory of Technique and Equipment for Macromolecular Advanced Manufacturing, South China University of Technology South China University of Technology, Guangzhou 510640, China; 2 Key Laboratory of Polymer Processing Engineering, Ministry of Education, South China University of Technology, Guangzhou 510640, China

**Keywords:** in-situ reaction, co-continuous structure, yield, debonding, crack propagation

## Abstract

In this study, the in-situ compatibilization reaction between recycled acrylonitrile–butadiene–styrene copolymer (rABS) and functional styrene–ethylene–butylene–styrene block maleic anhydride (SEBS-g-MAH) was confirmed, which contributed to the toughening phenomenon of rABS, especially the notched impact strength. As mechanical test that manifested, the rABS/SEBS-g-MAH blends are stronger and more ductile than the rABS/SEBS blends. Prominently, the former has great advantage over the latter in terms of improving the impact performance. Scanning electron microscope (SEM) images showed that the compatible segments that were generated by reaction not only improve the interface adhesion of rABS/SEBS-g-MAH blends but also promote the evolution of co-continuous structures, which can be evidently observed after etching. Furthermore, the SEM micrographs of tensile fracture surfaces indicated that the formation of the co-continuous phase and the improvement of interface adhesion are the most profound reasons for the excellent tensile properties of the rABS/SEBS-g-MAH blends. The impact fracture surface revealed that two-phase interface affects crack propagation and shear yielding absorbs more impact energy than simple interface debonding does at higher deformation rates. Meanwhile, rheological analysis demonstrated that the complex viscosity of the rABS/SEBS-g-MAH (80/20 wt%) blend with a co-continuous structure exhibits a maximum positive deviation at low frequencies from the theoretical value calculated using the rule of logarithmic sum, which indicated a connection between co-continuous structure and complex viscosity. In addition, the storage modulus vs. loss modulus curves of the blends revealed that the viscoelastic behavior of rABS/SEBS-g-MAH blends is very similar to that of rABS.

## 1. Introduction

Recycled plastic has been emphasized due to the increasingly scarce petroleum resources, as well as the gradually ascending demand of protecting environment [1,2,3]. Acrylonitrile–butadiene–styrene (ABS), which is a typical engineering polymer, has a huge market share in raw materials, in view of its excellent properties, such as outstanding notch impact resistance, high modulus caused by the benzene ring, and the oil resistance that is brought by the nitrile group [4,5,6]. Nevertheless, the repeated use of waste ABS has faced with a series of conundrums, including the loss of toughness, the decline in molecular weight, and the deterioration of surface quality, which has resulted in serious obstacles in the extensive application of recycled acrylonitrile–butadiene–styrene copolymer (rABS) [7,8].

In recent decades, the toughening modification of recycled plastics has been extensively studied [9,10]. Among these studies, it is generally thought that the microstructures have a significant effect on the mechanical properties of blends. Moreover, in-situ reactive compatibilization has been proved to be an efficient approach for reducing interfacial tension [11,12] and promoting the evolution of phase morphology of blends [13,14]. Besides, the performance of in-situ reactive compatibilization generally depends on the reaction between maleic anhydride (MAH) and –OH [15,16], and the reaction between glycidyl methacrylate (GMA) and –COOH or –OH [17,18]. In the blend with ABS as the main phase, generally the compatibilizer is compatible with ABS and react with another phase. For example, Liu et al. [19] added a core-shell impact modifier, styrene-maleic anhydride (SMA), in the rABS/polyamide (PA), which was successfully applied to enhance the interfacial adhesion between the ABS phase and PA phase. This is because the SMA is miscible with the styrene-acrylontrile (SAN) phase in ABS and the MAH group can react with PA. Similarly, the in-situ compatibilization reaction was applied to the rABS/wood-plastic composites (WPCs)/SMA blend [20]. Yeh et al. [20] suggested that the coupling agent (SMA) can effectively improve the mechanical properties of the blend due to the reaction of MAH with the –OH on the surface of cellulose. Such PA and cellulose, which contain functional chain-end groups, can offer the possibility of further chemical reaction. For this reason, the method for functionalizing the ABS was investigated. Fu et al. [21] prepared ABS-GMA by polymerizing the acrylonitrile, styrene, and GMA on polybutadiene particles, which can be utilized for improving the compatibility of the ABS/Poly (butylene terephthalate) (PBT) blend. However, adding the third component as a compatibilizer and grafting on the one of the polymer are both complicated and uneconomical. Under this scenarios, it is generally ignored that rABS can be oxidized to form a group, which can be applied to the in-situ reaction.

In previous reports, Shimade [22] and Adeniyi [23] have indicated that rABS will generate some oxygen-containing groups (hydroxyl, carbonyl, etc.) after thermal aging [24]. In this study, an experimental scheme of reactive compatibilization is designed on the basis of the fact that hydroxyl groups may be generated by rABS after oxidizing. The rABS/SEBSblends and rABS/SEBS-g-MAH blends are compared and analyzed while using mechanical test, infrared spectroscopy, scanning electron microscope observation, and rheological characterization. We mainly focus on the micro-morphology of the blends as well as the discrepancy in the crack propagation and fracture behavior between rABS/SEBS blends and rABS/SEBS-g-MAH blends, and discuss the possible reasons behind these phenomena. This is a new idea that the functional group of rABS itself can be used to compatibilize the rABS/SEBS-g-MAH blends.

## 2. Materials and Methods

### 2.1. Materials

Black rABS pellets with a melt flow index (MFI) that is equal to 1.63 g/10 min. (220 °C/2.16 kg) were supplied by Guangzhou Blonde Technology Co., Ltd. (Guangzhou, China). The commercial SEBS powder, Kraton G1651 (Kraton Corporation, Belpre, OH, USA) with an MFI less than 1 g/10 min. (230 °C/5 kg) and the styrene/ butadiene ratio of 30/70 was used in this study. SEBS-g-MAH, KratonFG1901 with an MFI of 22 g/10 min. (230 °C/5 kg) and the same styrene/ butadiene ratio, was purchased from Kraton Polymer Inc.(Belpre, OH, USA) The graft degree of maleic anhydride is about 1.4–2.0 wt%.

### 2.2. Blend Preparation

**Figure 1:**
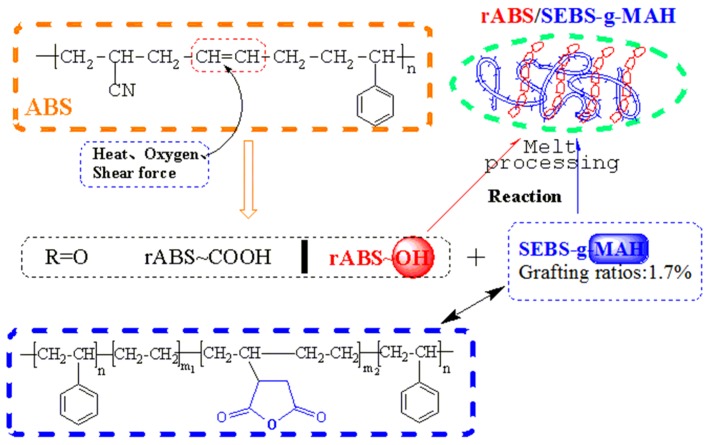
Schematic diagram of possible reaction routes in rABS/SEBS-g-MAH blend.

Prior to melt processing, rABS, SEBS, and SEBS-g-MAH were dried at 80 °C for 12 h, 8 h, and 8 h, respectively, in a convection oven. The rABS/SEBS and rABS/SEBS-g-MAH blends with different composition ratios (100/0, 95/5, 90/10, 85/15, 80/20 wt%) were first mixed by a high-speed mixer for 5 min. at room temperature. Subsequently, the melt blending of these premixed materials was performed by using a self-made triple screw extruder with a screw diameter of 25 mm, an aspect ratio of 40, a driving power of 75 kW, a maximum output of 30 kg/h, respectively. The temperature from the hopper to die was set as 150-170-190-200-200-200-200-195 °C, and the speed of screw was 80 rpm. Figure 1 shows the possible reaction routes. After extrusion, the extrudates were dried at 80 °C for 12 h in a convection oven. An injection molding machine (105GE, DongHua Machinery Co. Ltd., Dongguan, China) was utilized to prepare the standard dumbbell-shaped specimen based on ISO 527-2(type A) and rectangular specimens for the following mechanical test. The injection temperature from the hopper to nozzle was set as 190-200-210-210 °C, and the holding pressure, cooling temperature, and time are 3 MPa, room temperature, and 20 s, respectively.

### 2.3. Mechanical Testing

The uniaxial tensile properties of the blends were measured at room temperature while using an Instron 4302 universal tensile tester. All of the tests were conducted at a constant cross-head speed of 20 mm/min according to ISO 527-2 standards. The Izod impact strength of the blends with specimen dimension of 60 × 10 × 4 mm^3^ was tested while using an impact tester (Zwick5117, Zwick GmbH, Ulmer, Germany) according to ISO 180. The results presented are an average of at least five independent tests and the standard deviation.

### 2.4. Scanning Electron Microscopy (SEM)

The morphology of the blends was observed while using a Quanta FEG 250 SEM (Thermo Fisher Scientific, Hillsboro, OR, USA). The samples were cryo-fractured after immersing in liquid nitrogen. The SEBS phase was etched by xylene. All of the specimens, including the cryo-fracture surfaces, tensile fractured surfaces, and impact fractured surfaces, were coated with a thin layer of gold before observation.

### 2.5. Fourier Transform Infrared (FT-IR) Spectroscopy

The FT-IR equipment (Bruker vector 3, Bruker Optics, Karlsruhe City, Germany) was used to study the characteristic functional group reactions between rABS and SEBS-g-MAH. The samples were recorded at 32 consecutive scans and a resolution of 4 cm^−1^ within the wave numbers from 4000 to 400 cm^−1^.

### 2.6. Rheological Characterization Measurements

The rheological properties of the rABS/SEBS-g-MAH blends were studied while using a rotational rheometer (MCR 302, Anton Paar Gmbh, Graz, Austria) with the aim of evaluating the possible changes in viscoelastic behavior due to the in-situ reaction between rABS and SEBS-g-MAH. The viscosity-frequency scanning was performed under the protection of nitrogen at 210 °C, the scanning frequency was from 0.0628 to 628 rad/s, and the strain amplitude was kept at 1%.

## 3. Results and Discussion

### 3.1. Mechanical Properties

**Figure 2:**
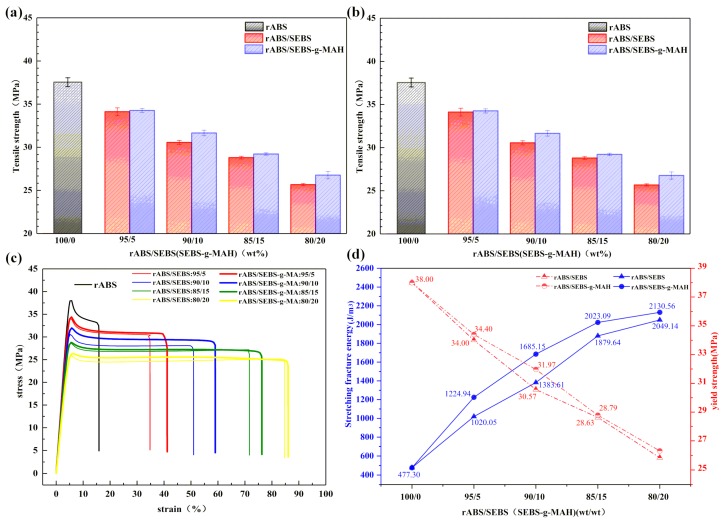
The tensile and impact properties of neat rABS, rABS/SEBS, rABS/SEBS-g-MAH blends: (**a**) impact strength; (**b**) tensile strength; (**c**) strain-stress curves; and, (**d**) fracture energy and yield strength.

Table 1 presents the results of the tensile and impact tests. It is common knowledge that the toughness will be increased, whereas the modules will be decreased when elastomer is added. However, Figure 2a shows that the impact strength of rABS/SEBS blends decreases with the addition of SEBS and the impact strength of rABS/SEBS blends is not sensitive to the content of SEBS. On the contrary, the impact strength of rABS/SEBS-g-MAH blends rises with the increase of the SEBS-g-MAH content, which shows a positive correlation. After adding 15 wt% SEBS-g-MAH, the impact strength of the blends starts to become higher than that of recycled ABS.

Under the condition of 20 wt% SEBS-g-MAH content, the impact strength of rABS/SEBS-g-MAH blend increases to 15.2 kJ/m^2^, which is higher than that of the recycled ABS and rABS/SEBS (80/20 wt%) blend by 35% and 88%, respectively. This particular phenomenon can be ascribed to the discrepancy of interface compatibility between the two blend systems. The improvement of interface compatibility between rABS and SEBS-g-MAH was achieved due to the in-situ compatibilization reaction between maleic anhydride and hydroxyl-terminated groups generated by oxidative degradation of rABS molecular chains in the processing process to ensure that SEBS-g-MAH can experience deformation and effectively absorb impact energy. SEM analysis and FT-IR analysis were conducted in the following sections in order to further study and confirm this conjecture. As shown in Figure 2b, the tensile strength decreases with the increase of the content of SEBS, whether the SEBS is grafted or not. However, it is worth noting that the rABS/SEBS-g-MAH blends are stronger and tougher than the rABS/SEBS blends (Figure 2c). As depicted in Figure 2d, the tensile fracture energy of the rABS/SEBS-g-MAH blends and the rABS/SEBS blends, i.e., the area under the stress strain curve, increases when the content of SEBS-g-MAH or SEBS increases, which is consistent with the dependence of the elongation at break on the content of SEBS-g-MAH or SEBS. The rABS/SEBS-g-MAH blends have better tensile toughness and yield strength than their counterpart. Therefore, the tensile toughness improvement of rABS/SEBS-g-MAH blends is not achieved through the reduction in the yield strength.

**Figure 3:**
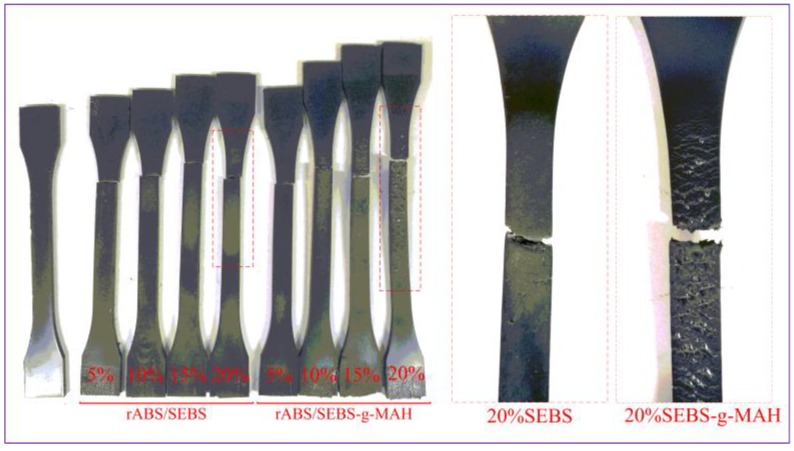
Photo of tensile sample.

As shown in Figure 3, both of the blend systems have obvious plastic deformation with the increase of the content of SEBS or SEBS-g-MAH. Large cracks and new surfaces can be observed on the surface of the rABS/SEBS-g-MAH blends after the tensile test, while the stress-whitening dominates in the rABS/SEBS blends. The new surfaces absorb certain deformation energy and the tensile toughness can be further improved, according to the theory of nonlinear fracture theory [25]. At the early stage of stretching, the elastomer particles first yield and deform, while the rigid plastic particles do not reach yield point. Subsequently, the two-phase interface will begin to generate voids. The strength of the interface determines the efficiency of stress transmission, and rABS/SEBS-g-MAH is undoubtedly even better. Good stress transfer between matrix and dispersed phase can promote deformation. SEM was used to observe the phase morphology and interfacial adhesion in order to investigate the main failure reasons of the two blend systems during tensile process.

### 3.2. Morphological Analysis

**Figure 4:**
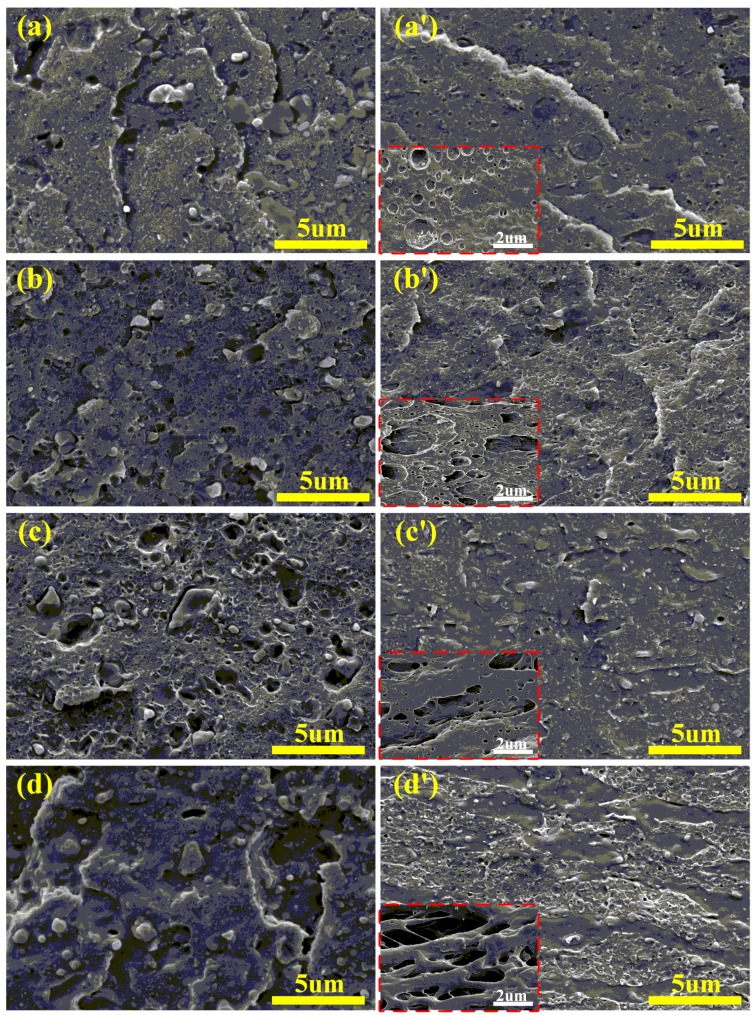
SEM micrographs of cryo-fracture of the rABS/SEBS blends and rABS/SEBS-g-MAH blends: (**a**) rABS/SEBS 95/5; (**a’**) rABS/SEBS-g-MAH 95/5; (**b**) rABS/SEBS 90/10; (**b’**) rABS/SEBS-g-MAH 90/10; (**c**) rABS/SEBS 85/15; (**c’**) rABS/SEBS-g-MAH 85/15; (**d**) rABS/SEBS 80/20; and, (**d’**) rABS/SEBS-g-MAH 80/20.

The effect of microstructure on polymer properties has been extensively studied [26,27,28]. It is widely accepted that morphological analyses, such as geometric analysis of dispersed phase and morphological analysis of the interface between matrix and dispersed phase, are helpful in analyzing the mechanical properties of polymer blends [29,30]. Figure 4 shows the SEM micrographs of cryo-fractured surfaces of the rABS/SEBS blends and rABS/SEBS-g-MAH blends. Additionally, the SEM micrographs of etched cryo-fractured surfaces of rABS/SEBS-g-MAH blends are shown in the insert of Figure 4a’–d’. Apparently, the compatibility of the rABS/SEBS blend is worse than that of the rABS/SEBS-g-MAH blend. For the same blend system, the microstructure of blend varies with the composition ratios of blend. In Figure 4a, the large SEBS phases with non-spherical shape are irregularly distributed in the matrix and there is an obvious interface between the rABS phase and SEBS phase. With the increase of SEBS content, the dispersed phase particles start to uniformly distribute in the matrix, but the compatibility between the rABS phase and SEBS phase is still poor. However, as clearly depicted in Figure 4a’, the rABS/SEBS-g-MAH blend has good compatibility with the matrix and its phase interface is quite fuzzy. It is because the in-situ compatibilization reaction plays the role of interfacial emulsification [31], which reduces the surface tension of the dispersed droplets and enables them to be better dissolved in the matrix. With the increase of the content of SEBS-g-MAH, the morphology of the dispersed phase has evolved into a shape with longer aspect ratio, such as ellipsoid, strip, and slice. Particularly, as shown in Figure 4d’, when the concentration of SEBS-g-MAH increases to 20 wt%, the blend makes great progress in the improvement of interfacial compatibility, which was indicated by the disappearance of the phase interface. The adhesion of the interface is also improved, which is consistent with the improvement of mechanical properties. In addition, the co-continuous structure was found in the rABS/SEBS-g-MAH blends containing 20 wt% SEBS-g-MAH.

**Figure 5:**
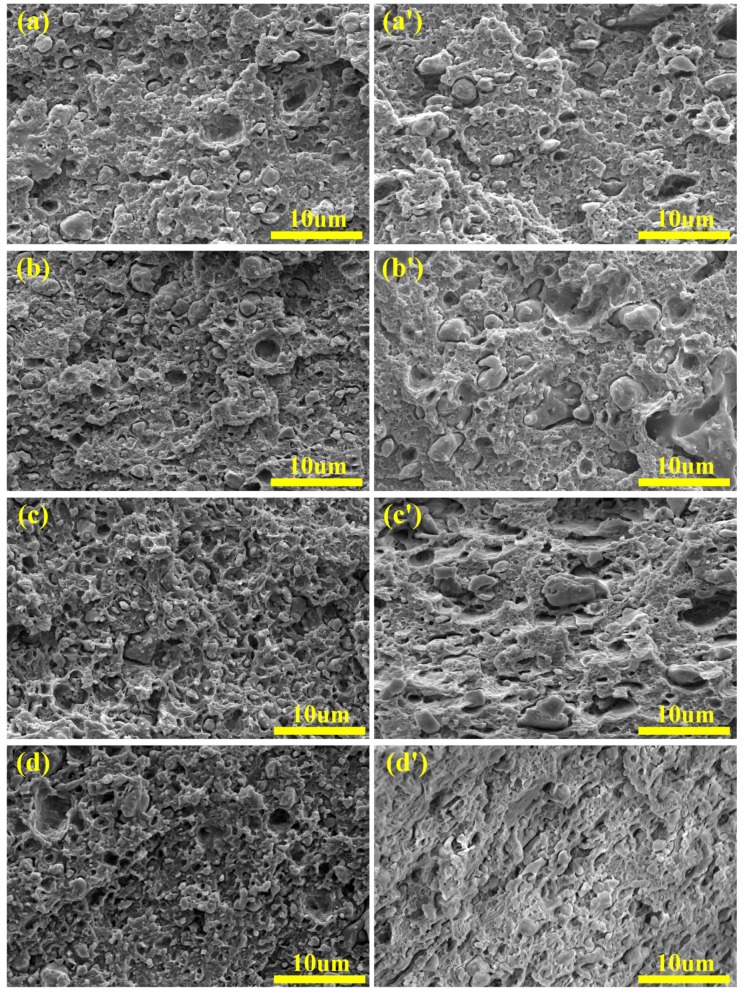
SEM micrographs of impact fracture surfaces of the rABS/SEBS blends and rABS/SEBS-g-MAH blends: (**a**) rABS/SEBS 95/5; (**a’**) rABS/SEBS-g-MAH 95/5; (**b**) rABS/SEBS 90/10; (**b’**) rABS/SEBS-g-MAH 90/10; (**c**) rABS/SEBS 85/15; (**c’**) rABS/SEBS-g-MAH 85/15; (**d**) rABS/SEBS 80/20; and, (**d’**) rABS/SEBS-g-MAH 80/20.

Figure 5 shows SEM micrographs of impact fracture surfaces of the rABS/SEBS blends and rABS/SEBS-g-MAH blends.

As shown in Figure 5a–d, the SEBS elastomer particles were seriously pulled out after impact. It is worth mentioning that, although the content of elastomer has increased, the debonding situation has not changed. The fracture surfaces show that the elastomer still retains a substantially ellipsoidal shape with severe voids between the two phases. In contrast, Figure 5a’–d’ show a small interfacial gap between the two phases and a significant deformation of the elastomer. In the rABS/SEBS-g-MAH (80/20 wt%) blend with the co-continuous structure (Figure 5d’), the two phases that penetrated each other exhibit consistent deformation. Therefore, for the rABS/SEBS blends, the main approach for releasing fracture energy is the rapid propagation of unstable cracks during the notch impact test, which cannot absorb a large amount of energy. Although SEBS acts as a stress concentrator, its induced microcracks rapidly expand into large cracks at the weak interface at a high deformation rate. Such unstable large cracks are hard to be terminated. Moreover, this phenomenon does not alter with the change of SEBS content. In other words, the increase of SEBS content has no effect on the impact performance of the rABS/SEBS blends, which is consistent with the experimental results of the impact strength test. Nevertheless, for the rABS/SEBS-g-MAH blends, the combination of interface is better than that of its counterpart, as shown in Figure 5. When the content of SEBS-g-MAH is below 20 wt%, the release of impact fracture energy was mainly attributed to the interfacial debonding and the deformation of SEBS-g-MAH. Therefore, the increase of SEBS-g-MAH content affected the improvement of impact toughness of the blends, which were attributed to the occurrence of the in-situ compatibilization reaction and the increase of the reaction degree [32]. What really makes a significant difference is the rABS/SEBS-g-MAH blend with 20 wt% SEBS-g-MAH. The elastomer in the rABS/SEBS-g-MAH blends has a short forced movement under shear stress due to the co-continuous structure and the great adhesion between the two phases, which suggests that partial impact fracture energy was consumed by the deformation of entire material, rather than only SEBS-g-MAH.

**Figure 6:**
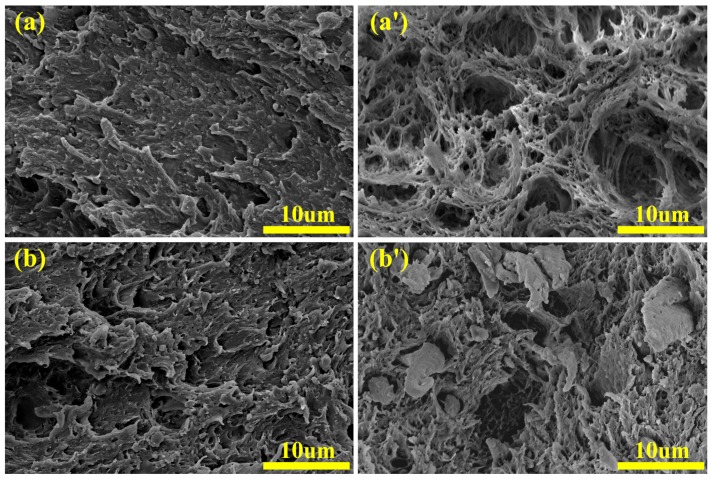

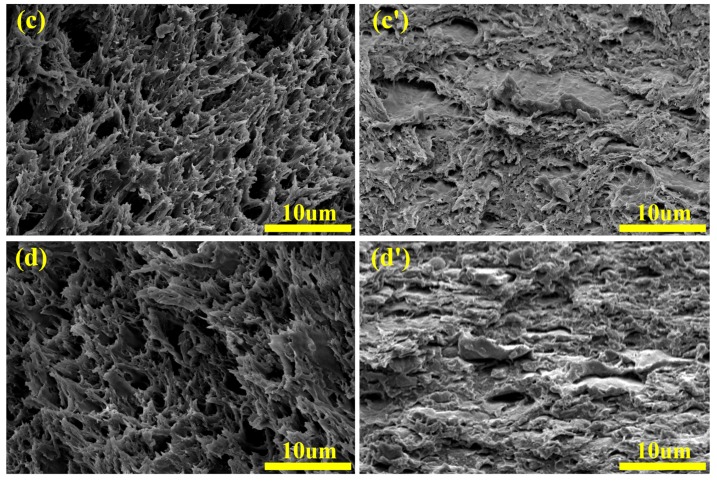
SEM micrographs of tensile fracture surfaces of the rABS/SEBS blends and rABS/SEBS-g-MAH blends: (**a**) rABS/SEBS 95/5; (**a’**) rABS/SEBS-g-MAH 95/5; (**b**) rABS/SEBS 90/10; (**b’**) rABS/SEBS-g-MAH 90/10; (**c**) rABS/SEBS 85/15; (**c’**) rABS/SEBS-g-MAH 85/15; (**d**) rABS/SEBS 80/20; and, (**d’**) rABS/SEBS-g-MAH 80/20.

**Figure 7:**
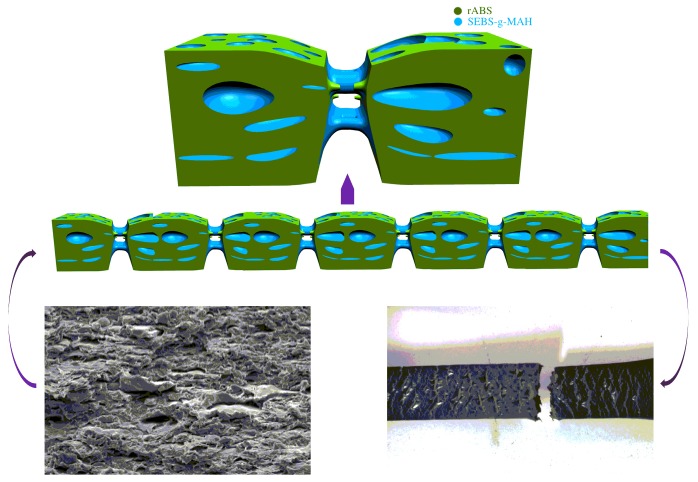
Fracture model of the tensile specimen of the rABS/SEBS-g-MAH (80/20) blend.

Figure 6 presents the SEM micrographs of tensile fracture surfaces of the rABS/SEBS blends and rABS/SEBS-g-MAH blends. As depicted in Figure 6a–d, all of the rABS/SEBS blends contain crazes that were highly oriented along the direction of the external force. With the addition of SEBS increasing, the distribution of crazes became denser. When it comes to the rABS/SEBS-g-MAH blends, deformation of SEBS-g-MAH can be clearly observed, in addition to the deformation of matrix (rABS). As shown in Figure 6a’, the crazes are of larger size than that in Figure 6a, it has better tensile toughness than the rABS/SEBS (95/5 wt%). As the addition of SEBS-g-MAH increased, bigger plastic deformation was observed in the rABS/SEBS-g-MAH blends. In previous mechanical analysis, it was known that the yield strength decreased, with the elastomer increasing not only in the rABS/SEBS blends, but also in the rABS/SEBS-g-MAH blends. Though the yield strength of the latter is higher than the former, the enhanced interfaces supported the bigger plastic deformation of SEBS-g-MAH, so the ductility of the latter are slightly higher than the former. Besides, the fracture mechanism of the rABS/SEBS-g-MAH (80/20 wt%) blend is likely to be the inconsistent critical shear conditions of rABS phase and SEBS-g-MAH phase when considering the large cracks on the surface of the tensile sample of the rABS/SEBS-g-MAH (80/20 wt%) blend and the co-continuous structure of the rABS/SEBS-g-MAH (80/20 wt%) blend shown in Figure 4d’. When subjected to a triaxial stress, the two phases begin to debond due to inconsistent deformation. Although the rABS phase has broken because its deformation goes beyond the elastic limit, the SEBS-g-MAH phase can continue to provide tensile strength, owing to the continuous structure (Figure 7). The failure occurs in the region where the co-continuous structure is not formed, such as the weak interface and defective area.

### 3.3. The FTIR Analysis

**Figure 8:**
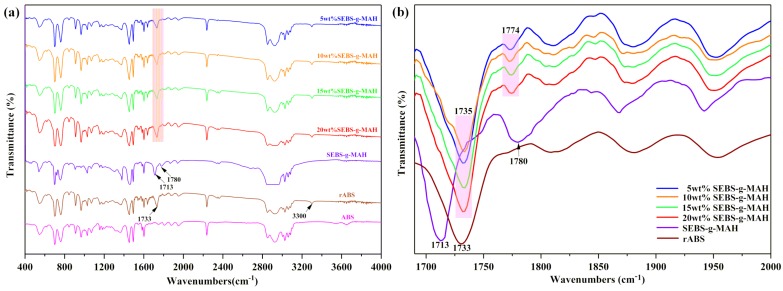
FT-IR spectra of neat ABS, neat rABS, neat SEBS-g-MAH and rABS/ SEBS-g-MAH blends: (**a**) wave numbers from 4000 to 400 cm^−1^, and (**b**) wave numbers from 2000 to 1680 cm^−1^.

Infrared spectroscopy has been considered to be an effective method for chemical structure characterization due to its high sensitivity as well as clear, fast and accurate detection of peaks [33]. The offset of characteristic absorption peaks of some functional groups provides extremely effective evidences for the occurrence of chemical reactions [34]. In this work, as demonstrated in Figure 8, it can be recognized that rABS has hydroxyl association peak at 3300 cm^−1^. This indicates that the existence of hydroxyl groups in rABS can react with MAH, which creates conditions for rABS blending modification. Moreover, a stretching vibration peak of carboxyl at 1733 cm^−1^ was observed at the infrared spectrum of the rABS curve, which corresponds to the results that oxidative degradation of carbon-carbon double bond in rABS will also generate other oxygen-containing groups, such as carboxyl, as reported in the literature [23,24]. Two strong absorption peaks of 1713 cm^−1^ and 1780 cm^−1^ were observed at the SEBS-g-MAH curve. The former is related with free carboxyl group and the latter is related to the stretching vibration of the carbonyl group of MAH [35]. The results suggested that the carbonyl peak in MAH was gradually weakened with the increase of SEBS-g-MAH content and there was no sharp strong peak in the rABS/SEBS-g-MAH blends as the content of SEBS-g-MAH was over 5 wt%. However, a red shift of the characteristic peak of carbonyl group to 1774 cm^−1^ has occurred. It is well known that the reaction of MAH with -OH will shift the absorption peak of carbonxyl to a low wavenumber, which was attributed to the destruction of cyclic structure in possession of high-energy anhydride groups. Meanwhile, the carboxy peak of the rABS/SEBS-g-MAH blends moved toward high wavenumber (1735 cm^−1^). This might be ascribed to the stronger polarity of R-COOH, which was the product of the in-situ reaction. A direct conclusion can be drawn that the in-situ reaction between rABS and MAH indeed exists although the relationship between reaction degree and the mass fraction of SEBS-g-MAH cannot be deduced merely from infrared spectrogram.

### 3.4. Rheological Properties

As is known, the microstructure of the material has significant effect on its rheological properties. Utracki and Sammut [36,37] hold that the viscosity of the blend and the sum of logarithm of the viscosity of each component have a positive deviation, which can indicate a strong interaction between the phases.

(1) $$\ln\eta_{blend}=\sum w_{i}\ln(\eta_{i}),$$

where: w*_i_* is the mass fraction of the *i*th component of the blend and η*_i_* is its viscosity [38].

The values have been obtained from the dynamic rheology according to the Cox–Merz rule:

η* = η_ss_(γ = ω), (2)

where: η* is the complex viscosity (Pa·s), η_ss_, steady shear viscosity (Pa·s), steady shear rate, frequency [39].

**Figure 9:**
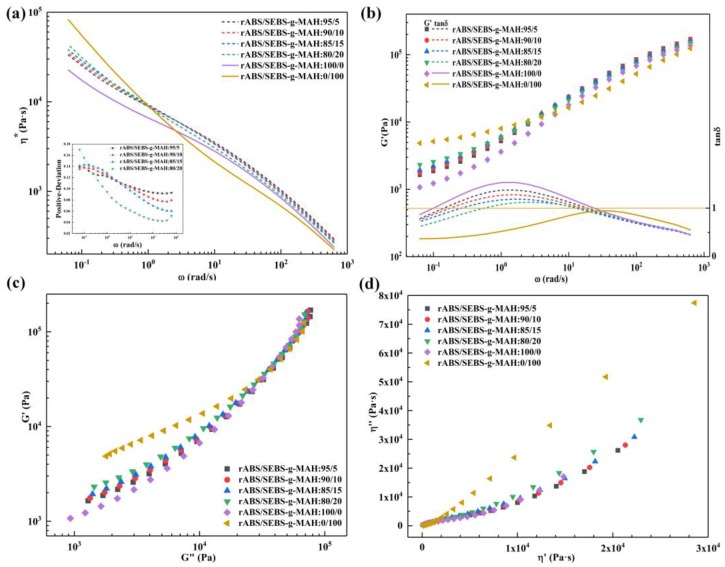
Rheological behavior of the rABS/SEBS-g-MAH blends at 210 °C: (**a**) Frequency dependence of η* and the positive deviation vs. ω; (**b**) Frequency dependence of G’ and tanδ; (**c**) the Han curves; and, (**d**) the Cole–Cole curves.

From Figure 9a, it can be concluded that the η* of rABS/SEBS-g-MAH blends was bigger than that of any pure component in the frequency range of 80~100 rad/s (for polymer processing). Furthermore, the statistical results show that the rABS/SEBS-g-MAH blends with different composition have a positive deviation. Noteworthy, the rABS/SEBS-g-MAH (80/20 wt%) blend exhibits a different deviant behavior. Firstly, its positive-deviation is the largest at low frequency, which might be attributed to the higher degree of in-situ reaction, the stronger interactions. Secondly, as the deviation sharply decreases with the increase of frequency, its deviation degree becomes the smallest of all rABS/SEBS-g-MAH blends finally. In a co-continuous model, the response of the two phases to strain is approximately equal, while the stress was transmitted through the continuous phase in the matrix-droplet model. On the one hand, in a co-continuous model, the blends undergo substantially the same external stress and the blends will exhibit high recoverable elasticity even if one phase (SEBS-g-MAH) is highly elastic and the other phase (recycled ABS) is not. On the other hand, even if the discontinuous phase (SEBS-g-MAH) is elastic in a matrix-droplet model, it is difficult to have large elastic recovery if the continuous phase is not elastic [40]. Consequently, as shown in the inset of Figure 9a, the rABS/SEBS-g-MAH (80/20 wt%) blend shows a unique positive deviation, owing to its high elastic recovery.

The rABS/SEBS-g-MAH blends can store more energy under external force due to the formation of new long branched-chain structures during the in-situ reaction. Therefore, as depicted in Figure 9b, increasing the content of SEBS-g-MAH results in a gradual raise of G’. The tanδ curve shows that the loss angle of the rABS/SEBS-g-MAH blends increases to a maximum value and then decreases. It is attributed to the lag and large internal friction of viscoelastic response in the intermediate frequency region [41]. In addition, with the increase of SEBS-g-MAH content, the loss peak moves to a high frequency, which indicates that the compatibility of the blend is also improved by the formation of more compatible segments. Particularly, viscoelastic transition points occur simultaneously at both low and high frequencies, as shown in Figure 9b. Moreover, the viscoelastic behavior of neat SEBS is dominated by the elastic response, which is determined by the distinctive structure of this thermoplastic elastomer [42]. Likewise, it can be observed that the viscoelastic properties of rABS/SEBS-g-MAH blends are still dominated by rABS, which is the main component of the blends.

The curves of the Han diagram can better illustrate this point [43]. It can be clearly seen from Figure 9c that the rheological behavior of the rABS/SEBS-g-MAH blends was close to that of rABS, but slightly shifted with the increase of SEBS-g-MAH content in the blends. In Figure 9d, The Cole–Cole graph does not show two relaxation arcs, which proves that the rABS/SEBS-g-MAH blends have good compatibility [44,45]. Notwithstanding, the Cole-Cole curve of the rABS/SEBS-g-MAH (80/20 wt%) blend still shows apparent dissimilarity from the remaining three curves. For one thing, the Cole–Cole curve of the rABS/SEBS-g-MAH (80/20 wt%) blend extends further away, which indicates a longer relaxation process. For the other thing, it is the only curve that all test points are higher than that of rABS curve, which can be ascribed to the peculiarity of SEBS-g-MAH phase morphology in the blend. The original rheological characteristics of continuous SEBS-g-MAH phase emerges, rather than being weakened on account of the discontinuous phase morphology.

## 4. Conclusions

In this work, the effect of maleic anhydride grafted SEBS on the properties of rABS/SEBS blends was investigated, especially the fracture phenomenon. FTIR results reveal that there are hydroxyl groups in rABS, which provides the possibility for in-situ compatibilization reactions. The rABS/SEBS-g-MAH blends show different mechanical properties, microstructure, and rheological behavior from the rABS/SEBS blends. Through the analysis of mechanical properties, we found that the rABS/SEBS-g-MAH blends have more excellent tensile strength, ductility, and impact resistance than rABS/SEBS blends and rABS do. Through the analysis of microstructure, a conclusion can be drawn that the good phase interface and the co-continuous structure are the fundamental reasons for the improvement of mechanical properties of the rABS/SEBS-g-MAH blends. The observation of SEM micrographs of impact fracture surfaces demonstrates that simple debonding cannot provide strong assistance for impact modification, whereas stronger interface and matrix deformation are the effective ways of absorbing a lot of impact energy. The SEM micrographs of the tensile fracture surfaces prove that crazes and shear band deformation can all contribute to the improvement of ductility during the large deformation stage of the amorphous polymer. The rABS/SEBS-g-MAH (80/20 wt%) blend with co-continuous structure exhibits unique failure phenomenon that stretching of elastomer makes further contribution to the improvement of ductility. Rheological behavior further confirmed that a co-continuous phase structure was formed when 20 wt% SEBS-g-MAH was added. The rheological performance of the rABS/SEBS-g-MAH blends was disturbed by the long branched-chain structure that was generated in the in-situ compatibilization reaction. Moreover, the viscoelastic properties of rABS/SEBS-g-MAH blends were close to that of rABS due to the dominance factor of component composition. Ultimately, the primary reasons for toughening of rABS are proposed, which provides guidance for the toughening modification of rABS and it is beneficial in better understanding the fracture failure phenomenon.

## Figures and Tables

**Table 1:** The mechanical properties of neat rABS, rABS/SEBS, and rABS/SEBS-g-MAH blend.

Sample	Tensile Strength (MPa)	Modulus of Tensile (MPa)	Elongation at Break (%)	Impact Strength (kJ/m^2^)	Fracture Energy (J/m^3^)
rABS	37.5 ± 0.5	845.1 ± 25.9	15.8 ± 2.6	11.3 ± 0.9	477.3
rABS/SEBS:95/5	34.1 ± 0.5	807.6 ± 11.7	34.0 ± 5.3	8.1 ± 0.4	1020.1
rABS/SEBS:90/10	30.6 ± 0.2	748.0 ± 8.7	49.6 ± 10.8	8.1 ± 0.4	1383.0
rABS/SEBS:85/15	28.8 ± 0.2	679.3 ± 18.7	71.9 ± 8.7	8.0 ± 0.5	1880.6
rABS/SEBS:80/20	25.7 ± 0.1	625.2 ± 5.7	84.4 ± 5.0	8.1 ± 0.6	2049.1
rABS/SEBS-g-MAH:95/5	34.3 ± 0.3	805.2 ± 6.7	41.4 ± 4.3	9.5 ± 0.5	1225.9
rABS/SEBS-g-MAH:90/10	31.7 ± 0.3	736.8 ± 8.8	57.6 ± 6.7	10.0 ± 0.1	1685.2
rABS/SEBS-g-MAH:85/15	29.2 ± 0.1	681.9 ± 5.6	76.2 ± 2.8	11.8 ± 0.7	2023.1
rABS/SEBS-g-MAH:80/20	26.8 ± 0.4	608.9 ± 7.1	85.8 ± 4.5	15.2 ± 0.8	2131.6
